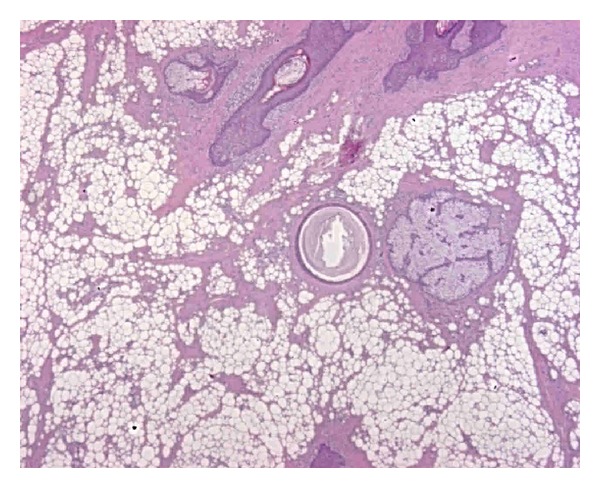# Penile Subcutaneous Fibrolipoma Postaugmentative Phalloplasty

**DOI:** 10.1155/2013/696314

**Published:** 2013-09-30

**Authors:** Patrizio Vicini, Ferdinando De Marco, Piero Letizia, Lavinia Alei, Gabriele Antonini, Giovanni Alei, Vincenzo Gentile

**Affiliations:** ^1^Department of Urology, Italian Neurotraumatologic Institute Grottaferrata “I.N.I.”, 00046 Grottaferrata, Rome, Italy; ^2^Department of Plastic Surgery, “Sapienza” Rome University, Rome, Italy; ^3^Department of Dermatology, “Sapienza” Rome University, Rome, Italy; ^4^Department of Urology, “Sapienza” Rome University, Rome, Italy

## Abstract

Fibrolipomas are a rare subtype of lipomas. We describe a case of a man suffering from subcutaneous penile fibrolipoma, who three months earlier has been submitted to an augmentative phalloplasty due to aesthetic dysmorphophobia. After six months from the excision of the mass, the penile elongation and penile enlargement were stable, and the patient was satisfied with his sexual intercourse and sexual life. To our knowledge, this is the first reported penile subcutaneous fibrolipoma case in the literature. The diagnostics and surgical features of this case are discussed.

## 1. Introduction

As described in literature, penis size is an issue of social relevance [1, 2].

Some men who were considered normal may request augmentation surgery as a result of an altered perception of the size of the organ (penile dysmorphophobia); in this case, dysmorphophobia may represent a functional problem (a patient with normal penis but dissatisfied with its size during erection) or aesthetic problem (a patient whose penis is normal but who is dissatisfied with its dimension in flaccid state) [3–5]. Most of elongation and enlargement phalloplasty operations have a more apparent effect rather than real effect, rising an increase of the penile size that is more evident during flaccid state rather than during erective status, these procedures are indicated exclusively for patient with aesthetic dysmorphophobia [3–6].

In these patients, a simple operative procedure can be used to enlarge the volume of the penis by autologous fat transfer; at the same time, the penis can be lengthened by the release of the suspension ligament below the symphysis, so defined “augmentative phalloplasty” [7–10].

We describe a case of subcutaneous fibrolipoma in a man submitted in the previous three months to augmentative phalloplasty due to aesthetic dysmorphophobia, to the knowledge of the researchers this is the first case reported in the literature.

## 2. Case Report

We describe the case of a 54-year-old man who presented small penis. After urological consult, he agreed to undergo an augmentative phalloplasty resulting in penis elongation and enlargement. The procedure was performed on April 2012 under general anaesthesia. After skin incision at the base of the penis, a dissection of the ligament suspensory of penis below the symphysis was done, extracting the corpus cavernosus, and anchoring the tunica albuginea at the periost. At the root of the phallus, the skin was elongated by V-Y plasty.

Following that, the sovrapubic liposuction was performed. Fractions of fat were centrifuged, purified of serum and oil, and put into 2 mL syringes.

Incision with a scalpel at the stretched preputium between the two forceps was done.

By inserting and pulling back the Coleman Cobra cannula, a purified body fat was transplanted from the penis root upwards, positioning longitudinal steri strips at the end of surgery. Overall 40 mL of purified fat cells was transferred.

At the end of procedure, the patient obtained an increase of 4 cm in length and 2,5 cm in circumference. Before surgery, the length of the penis in flaccid state was 4,5 cm, immediately after surgery the length of the penis increased to 8,5 cm. Before augmentation with autologous fat transfer, the circumference of the penis in flaccid state was 8 cm, after augmentation with autologous fat transfer the circumference of the penis was 10,5 cm.

Patient was advised to refrain from sexual activity for 5 weeks after the surgery.

Three months later, the man was found to have a subcutaneous dorsal penile mass. 

**Figure 1:**
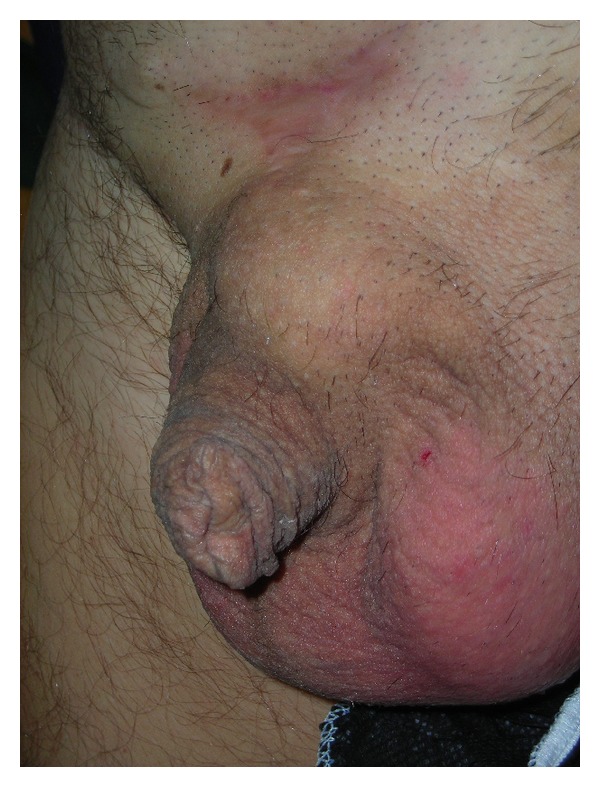


This mass caused pain during erection and difficulty in having a sexual intercourse because of the limited penile extension during erection such as trapped penis (Figure 1).

During penile examination, a mass of approximately 6 × 6 × 4 cm was palpated at the basis of penis, at the level of the proximal third of the penile shaft, on the dorsal surface of the organ. 

On palpation, the mass was firm in consistency, painless, and mobile under the palpating finger.

No abnormalities were detected in the glans and other organs. The patient did not complain about any other medical problems. 

Ultrasonography of the mass was performed using B-mode equipment (Sonoscape S8, Milestone Company) and a linear 8.0 MHz transducer (L741, Milestone Company). Ultrasonography showed a structure with a loss of homogenous echogenicity with multiple hyperechoic areas. 

We waited further six months after surgery to assess the possible spontaneous retraction of the mass, but no changes were observed so we decided to remove it. 

Under general anesthesia, an infrapubic incision at the base of penis was performed to excise the mass; it was dissected from the lateral subcutaneous tissue easily, but it was very difficult to dissect it from the below penile neurovascular bundle.

**Figure 2:**
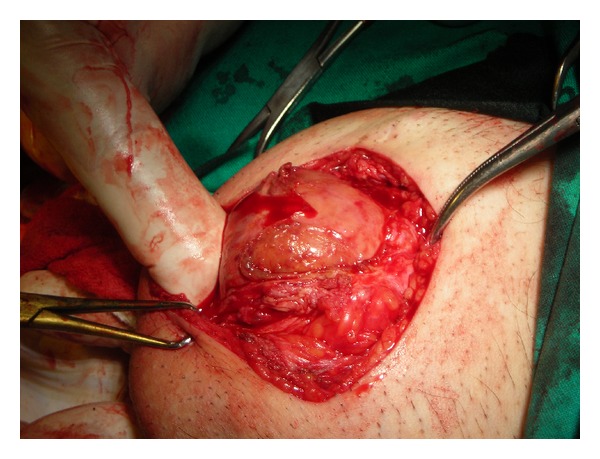


The mass (6 × 6 × 4 cm) had an elastic texture and was enclosed by a smooth, shining, and vascular capsule (Figure 2). On the cut surface, a large amount of the interior of the mass was milky-white and composed of homogeneous adipose-like tissue. 

The excised tissue was sent for histological examination.

Tissue samples of the mass were fixed in 10% buffered formalin and processed for paraffin embedding as standard procedure.

The sections were deparaffinised, dehydrated, and stained with haematoxylin and eosin. Microscopic histopathological examination of the mass demonstrated adipose tissue with diffusely distributed mature adipocytes and septa of fibrous connective tissue with proliferating fibroblast (Figure 3).

On the basis of these clinical and histologic findings, a diagnosis was made of a subcutaneous fibrolipoma of penis.

There were no postoperative complications. After six months from the second surgery, the penile elongation and penile enlargement were stable, and the patient was satisfied with his sexual intercourse and sexual life.

The satisfaction of surgical outcome was also assessed at 12 months following the surgery by directly asking the patients “are you satisfied with the result of your surgery?” 

The patient was satisfied with the outcome of the surgery.

At the 12th month, during the postoperative follow-up visit, the patient reported a normal erectile function; overall, the ED domain score was 25, and the Rigiscan test revealed a good number of nocturnal penile erections (3-4 a night), with a normal duration and a good rigidity both at the base and at the tip of penis (>70%). An artificial erection, induced with the administration of 10 mcg of prostaglandin E, ruled out the presence of any curvature.

## 3. Discussion

To the best of the authors' knowledge, this is the first report of subcutaneous penile fibrolipoma in man. 

Fibrolipoma is a histological variant of lipoma, the aetiology of this condition is not well understood, usually in other organs this lesion is an uncommon benign tumour [11, 12]. 

The consistency of the fibrolipoma can be soft or firm depending on the amount of fibrous tissue, and the treatment is usually an excision [13].

The condition can occur in the oral cavity, subcutaneous tissue, colon, trachea, larynx, parotid gland, and spermatic cord as a slow growing lesion [13].

As reported in the literature, in men affected by aesthetic dysmorphophobia, a simple operative procedure can be used to enlarge the volume of the penis by autologous fat transfer. At the same time, the penis can be lengthened by the release of the suspension ligament below the symphysis, so defined as “augmentative phalloplasty” [7–10].

Probably bleeding and lymphorrhoea after surgery, together with the amount of fat transferred, could play a role in the aetiology of penile fibrolipoma such as in our case.

In our opinion, it could be useful to perform sovrapubic liposuction, autologous fat transfer, and dissection of suspension ligament separately, so to reduce the amount of bleeding.

We suggest even to use a compressive bandage on the sovrapubic area for 4–6 weeks and to reduce the amount of fat transferred during the procedure.

For the diagnosis of the lesion, we effectuated only clinical palpation and ultrasonography.

In addition to that in the patient's preoperative diagnosis, a magnetic resonance imaging (MRI) should be performed.

As reported in the literature, MRI could be very useful not only for the diagnosis of all kinds of lipomas, but also for the connection with the neighboring structures [14]. 

In our patient, surgical resection of the mass was technically difficult because of the near connection with penile neurovascular bundle and the adherences to it. Most probably, the MRI could have helped us to provide better understanding of the involvement of local structures. 

Augmentative phalloplasty with autologous fat transfer and section of the suspension ligament below the symphysis is a generally safe and effective procedure.

Occasional complications are possible even with a meticulous observation of the steps of the operation.

Further investigation is warranted to clarify the aetiology of this subcutaneous penile fibrolipoma after augmentative phalloplasty.

The complete resection of the mass, consisting in surgical excision with capsular dissection, should be performed but it is not always easy.

MRI increases the accuracy of diagnosis and helps to determine the best surgical approach.

## Figures and Tables

**Figure 3:**